# CLSI-Derived Hematology and Biochemistry Reference Intervals for Healthy Adults in Eastern and Southern Africa

**DOI:** 10.1371/journal.pone.0004401

**Published:** 2009-02-06

**Authors:** Etienne Karita, Nzeera Ketter, Matt A. Price, Kayitesi Kayitenkore, Pontiano Kaleebu, Annet Nanvubya, Omu Anzala, Walter Jaoko, Gaudensia Mutua, Eugene Ruzagira, Joseph Mulenga, Eduard J. Sanders, Mary Mwangome, Susan Allen, Agnes Bwanika, Ubaldo Bahemuka, Ken Awuondo, Gloria Omosa, Bashir Farah, Pauli Amornkul, Josephine Birungi, Sarah Yates, Lisa Stoll-Johnson, Jill Gilmour, Gwynn Stevens, Erin Shutes, Olivier Manigart, Peter Hughes, Len Dally, Janet Scott, Wendy Stevens, Pat Fast, Anatoli Kamali

**Affiliations:** 1 Projet San Francisco (PSF), Kigali, Rwanda; 2 Uganda Virus Research Institute (UVRI), Entebbe, Uganda; 3 Kenya AIDS Vaccine Initiative (KAVI), Nairobi, Kenya; 4 MRC/UVRI Uganda Virus Research Unit on AIDS, Masaka Site, Masaka, Uganda; 5 Zambia Emory HIV Research Project (ZEHRP), Lusaka, Zambia; 6 Centre for Geographic Medicine Research-Coast (CGMRC), Kenya Medical Research Institute (KEMRI), Kilifi, Kenya; 7 Emory University, Atlanta, Georgia, United States of America; 8 International AIDS Vaccine Initiative - New York, New York, United States of America; 9 Centre for Tropical Medicine, Nuffield Department of Clinical Medicine, University of Oxford, Oxford, United Kingdom; 10 The EMMES Corporation, Rockville, Maryland, United States of America; 11 University of the Witwatersrand, Johannesburg, South Africa and National Health Laboratory Services, Johannesburg, South Africa; 12 Johnson and Johnson, New Brunswick, New Jersey, United States of America; 13 University of Oxford, Oxford, United Kingdom; 14 El Cerrito, California, United States of America; University of Louisville, United States of America

## Abstract

**Background:**

Clinical laboratory reference intervals have not been established in many African countries, and non-local intervals are commonly used in clinical trials to screen and monitor adverse events (AEs) among African participants. Using laboratory reference intervals derived from other populations excludes potential trial volunteers in Africa and makes AE assessment challenging. The objective of this study was to establish clinical laboratory reference intervals for 25 hematology, immunology and biochemistry values among healthy African adults typical of those who might join a clinical trial.

**Methods and Findings:**

Equal proportions of men and women were invited to participate in a cross sectional study at seven clinical centers (Kigali, Rwanda; Masaka and Entebbe, Uganda; two in Nairobi and one in Kilifi, Kenya; and Lusaka, Zambia). All laboratories used hematology, immunology and biochemistry analyzers validated by an independent clinical laboratory. Clinical and Laboratory Standards Institute guidelines were followed to create study consensus intervals. For comparison, AE grading criteria published by the U.S. National Institute of Allergy and Infectious Diseases Division of AIDS (DAIDS) and other U.S. reference intervals were used. 2,990 potential volunteers were screened, and 2,105 (1,083 men and 1,022 women) were included in the analysis. While some significant gender and regional differences were observed, creating consensus African study intervals from the complete data was possible for 18 of the 25 analytes. Compared to reference intervals from the U.S., we found lower hematocrit and hemoglobin levels, particularly among women, lower white blood cell and neutrophil counts, and lower amylase. Both genders had elevated eosinophil counts, immunoglobulin G, total and direct bilirubin, lactate dehydrogenase and creatine phosphokinase, the latter being more pronounced among women. When graded against U.S.-derived DAIDS AE grading criteria, we observed 774 (35.3%) volunteers with grade one or higher results; 314 (14.9%) had elevated total bilirubin, and 201 (9.6%) had low neutrophil counts. These otherwise healthy volunteers would be excluded or would require special exemption to participate in many clinical trials.

**Conclusions:**

To accelerate clinical trials in Africa, and to improve their scientific validity, locally appropriate reference ranges should be used. This study provides ranges that will inform inclusion criteria and evaluation of adverse events for studies in these regions of Africa.

## Introduction

Clinical trials are increasingly being conducted in Africa, especially trials of preventive interventions for HIV, tuberculosis and malaria. Great strides have been made towards improving the research infrastructure worldwide, especially in Africa [1], [2]. However, laboratory reference intervals used for trial screening and evaluating adverse events (AE) have often been derived from predominantly North American and European (largely Caucasian) populations [3]; use of these reference intervals may lead to unnecessary exclusion of potential participants.. Previous studies from Eastern and Southern African populations indicate differences in hematology and immunology values, including lower values for hemoglobin, hematocrit, red blood cell count (RBC), platelets, mean corpuscular volume (MCV) [4], [5], [6], [7], [8], [9] and neutrophils and increased values for monocytes and eosinophils [5], [7], [9], [10], [11]. Lymphocyte and CD4 T cell counts in Africans have also been reported to be lower than intervals measured in Europe and North America [9], [12], [13]. Other studies have noted hematology and CD4 T cell count variations across different regions of Africa [5], [9], [14]. Within the U.S., lower neutrophil and leukocyte counts have been found to be more common among blacks relative to whites [15]. To date, no studies have assessed laboratory reference intervals in a controlled, systematic manner across multiple African sites among asymptomatic adults who would otherwise be eligible as healthy clinical trial volunteers. Locally appropriate reference intervals are essential for planning and executing trials in a safe, efficient and ethical manner.

This paper presents the results from a cross sectional study in seven African research facilities to: 1) establish values for locally relevant serum chemistry and hematology analytes among healthy African adults in anticipation of future clinical trials of HIV prevention technologies and other interventions, 2) compare these findings to established intervals from the U.S., and 3) determine how many individuals would have been reported as having an adverse event (AE) according to the DAIDS AE Grading Table [16].

## Methods

### Ethical Considerations

This study was approved by the National Ethics Committee of Rwanda, the Uganda Virus Research Institute Science and Ethics Committee, the Uganda National Council for Science and Technology, the Kenya Medical Research Institute Ethics Committee, Kenyatta National Hospital Ethics and Research Committee at the University of Nairobi, the University of Zambia Biomedical Research Ethics Committee and the Emory University School of Public Health Ethics Committee. All EC/IRBs are registered with the U.S. Office of Human Research Protection. Written informed consent was obtained prior to conducting any study procedures, literacy was not a requirement for participation.

### Reference populations

Healthy male and female volunteers aged 18–60 years with a documented HIV-negative test in the prior four weeks were screened and enrolled across seven clinical research centers in four countries (Kigali, Rwanda; Masaka and Entebbe, Uganda; Kangemi and Kenyatta National Hospital [KNH] in Nairobi and Kilifi, Kenya; and Lusaka, Zambia). The methods have previously been described in detail [17]. Briefly, 200 to 400 volunteers were recruited at each site with equal numbers of men and women represented by design. Volunteers were largely recruited from new or ongoing cross-sectional and prospective cohort studies conducted in preparation for future HIV vaccine safety and efficacy trials. Potential volunteers were screened out if a medical history revealed that they were acutely ill, had significant recent illness, or were pregnant. Menstruating women were invited to return in two weeks. Breastfeeding women were not excluded. At enrollment, demographic information and medical history were collected, a physical examination was performed, and blood and urine samples were collected. HIV counselling and testing was provided if the volunteer did not have a documented negative HIV test performed in the previous four weeks. Enrolled volunteers were excluded from the study analysis if they had significant findings on physical examination or if laboratory tests revealed that they were pregnant, HIV antibody positive, had evidence of hepatitis B or C infection or suspected syphilis.

### Laboratory

Blood was tested for Hepatitis B surface antigen (Abbot-Murex HBsAG ELISA version 3 or Biomerieux Hepanostika HBsAg Uni-Form II MicroELISA system), Hepatitis C antibody (Abbot-Murex Anti-HCV version 4 or Innogenetic Innotest HCV Ab IV), HIV antibodies [17] and RPR serostatus. Urine pregnancy tests for hCG were performed on all women. Blood was tested for 25 analytes including clinical chemistry, hematology (complete blood count, with 5-part automated differential and platelet count), and CD4 and CD8 T cell count. All laboratories used the Beckman Coulter AcT 5 diff CP Hematology Analyzer (Beckman Coulter, USA) and the Vitalab Selectra E Clinical Chemistry Analyzer (Vital Scientific, The Netherlands). Four clinical centers (Kangemi, KNH, Entebbe, and Masaka) did not to perform alkaline phosphatase (ALP) testing with the standardized reagents; these data are not shown. Five laboratories used the FACSCount System (BD Biosciences, USA), while the KAVI laboratory used the FACSCalibur (BD Biosciences, USA). All machines were calibrated to record CD4 values ≥2,000 cells/µL as 2,000 cells/µL. To minimize CD4 diurnal variation, samples were drawn before noon. Specimens with low CD4 counts (<350 cells/µL) were tested for HIV RNA by polymerase chain reaction (PCR, Roche Amplicor) to rule out antibody-negative, acute HIV infection.

All laboratory staff received equipment training and were required to pass an independent quality review before enrolling volunteers. Analyte results were validated throughout the course of the study through a central reference laboratory (Central Laboratory Services [CLS], Johannesburg, South Africa) external quality assurance (EQA) program provided by the South African National Health Laboratory Services [18], [19]. Results were compared across technicians and across laboratories. The study was conducted according to the principles of Good Clinical Laboratory Practices [20].

### Data collection and analysis

Data were directly recorded on Case Report Forms which were then faxed to a central server using DataFax software (Clinical DataFax Systems Inc., Hamilton, Canada). Data analyses were conducted using Stata (v9.1 College Park, TX, USA) and SAS (v9.1, Cary, NC, USA) software. The Clinical and Laboratory Standards Institute (CLSI, www.clsi.org, formerly NCCLS) terms and guidelines for defining reference intervals [21] were followed. Briefly, we evaluated intervals by clinical centers and gender. If the overall F-test from an ANOVA on mean values was statistically significant (p<0.05), a step-wise procedure was performed to evaluate which intervals were similar enough to combine into a ‘study consensus interval.’ For parameters that were long-tailed, all ANOVA tests were performed after a log transformation and geometric means were compared instead of the arithmetic means. First, we compared the two most similar sites' intervals based on the p-values obtained from the overall ANOVA, which were adjusted for multiple comparisons using the Tukey method. If not statistically significantly different, then the data from the two sites were combined. If significantly different, but the difference between means was less than 25% of the width of the 95% reference interval estimated from the combined sample, and the ratio of standard deviations was less than 1.5, then the data from the two sites were combined. The combined data were then compared to each remaining site as described above in a new ANOVA. This was repeated until all sites were combined into or excluded from the study consensus interval. Finally, the study consensus intervals for men and women were compared as above, and the data combined if differences were not significant, as described above. Study consensus intervals are shown as the 2.5^th^ and 97.5^th^ percentiles between which lies 95% of our reference sample group data.

Study consensus intervals were first compared to U.S.-derived laboratory intervals from the Massachusetts General Hospital (MGH) [22] for most analytes, from *Bakerman*'*s ABCs of Interpretive Laboratory Data* [23] for white blood cell differentials (which are not presented in the MGH data), and from the Becton Dickson FACSCount package insert for CD4 and CD8 T cell counts. Collectively, these U.S.-derived laboratory intervals will be referred to as the “U.S.-based comparison intervals.” Because these published comparison intervals do not provide details on laboratory methodology, inclusion/exclusion criteria, sample sizes or standard errors, statistical confirmation in comparing laboratory intervals was not possible. We present the number and percent of volunteers in our study with out-of-range (OOR) values when compared to the U.S.-based comparison intervals.

We then applied the AE grading criteria provided by Division of AIDS, National Institute of Allergy and Infectious Diseases, of the U.S. National Institutes of Health (DAIDS) [16] to determine how many study volunteers would have been classified as having an AE. These criteria consider 12 of the analytes presented in this report. The DAIDS AE grading cutoffs for hematology are absolute numbers, while the chemistry cutoffs are relative to the “normal” limits of a particular laboratory, site, or population. For the latter, we applied the DAIDS AE criteria to the U.S.-based comparison intervals described above in order to create the DAIDS-based AE grading cutoffs for chemistries. We present the number and percent of volunteers in our study who would have been classified as an AE when using the U.S.-derived DAIDS AE grading criteria.

## Results

### Study population

From December 2004 to October 2006, a total of 2,990 potential volunteers were screened, of whom 2,387 (80%) were enrolled and 2,105 (70%) were included in the final analysis. A detailed report of the study population recruitment, enrollment and exclusions has been published [17]. Volunteers who were screened out by history and physical examination after informed consent but prior to enrollment tended to be older than those enrolled (median age: 30 vs. 28 years, respectively, p = 0.001) and were more likely to be female (22.8% vs. 17.6% males, p<0.001). Of those enrolled, 282 (12.0%) were excluded from analysis, mainly due to a positive test for HBsAG (106, 40%), Hepatitis C antibody (95, 36%), or a positive RPR (55, 21%). More males were excluded from analysis, although the difference was not statistically significant (12.5% vs. 9.4% females, p = 0.06). For a listing of all values by clinical center and by gender, the complete study report may be found online (www.iavi.org/protocolD), or contact the corresponding author.

The final analysis cohort of 2,105 comprised 1,022 (49%) women and 1,083 (51%) men. The age range was 18–59 years (median: 28) and varied by location (Table 1). The highest level of education achieved also differed by location. The KNH cohort primarily included medical and health professionals; the other sites had more generalized urban and rural populations. Nearly 20% of the Kigali cohort reported completing no formal education. The majority of volunteers reported no alcohol use; only 94 (4.5%) reported daily intake of alcoholic beverages. The greatest number of smokers was reported in Kangemi (34%) and the lowest in Entebbe (<2%). Reported recreational drug use was very low, and 94% of the cohort reported no use.

**Table 1:** Characteristics of reference sample groups by location

	Total		Kigali, Rwanda		Kangemi, Kenya		Kenyatta National Hospital, Kenya		Entebbe, Uganda		Masaka, Uganda		Lusaka, Zambia		Kilifi, Kenya	
	N	%	N	%	N	%	N	%	N	%	N	%	N	%	N	%
Average altitude (m)	NA		1567		1680		1680		1132		1321		1300		10	
Total	2105		373		362		197		194		331		352		296	
Age (years)																
Median	28		28		30		23		25		27		30		27	
Range	18–59		18–53		18–58		18–59		18–55		18–58		18–58		18–55	
Body Mass Index (kg/m^2^)																
Median	21.2		21.1		21.6		21.4		21.8		21.0		20.4		21.5	
95 %ile°	17.3–31.3		17.3–29.6		16.6–32.7		17.0–34.8		18.5–30.5		18.0–28.6		17.0–31.5		16.9–31.2	
Gender																
Male	1083	51.4	185	49.6	186	51.4	98	49.7	96	49.5	183	55.3	168	47.7	167	56.4
Female	1022	48.6	188	50.4	176	48.6	99	50.3	98	50.5	148	44.7	184	52.3	129	43.6
Highest Education																
None	161	7.6	71	19.0	18	5.0	1	0.5	2	1.0	29	8.8	12	3.4	28	9.5
Primary	1088	51.7	264	70.8	224	61.9	26	13.2	26	13.4	261	78.9	141	40.1	146	49.3
Secondary	597	28.4	30	8.0	111	30.7	64	32.5	100	51.5	38	11.5	157	44.6	97	32.8
>Secondary	237	11.3	3	0.8	6	1.7	104	52.8	57	29.4	2	0.6	42	11.9	23	7.8
Missing	22	1.0	5	1.3	3	0.8	2	1.0	9	4.6	1	0.3	0		2	0.7
Smoker																
No	1757	83.5	319	85.5	239	66.0	171	86.8	191	98.5	305	92.1	305	86.6	227	76.7
Yes	348	16.5	54	14.5	123	34.0	26	13.2	3	1.5	26	7.9	47	13.4	69	23.3
Alcohol Use																
None	1334	63.4	215	57.6	175	48.3	133	67.5	166	85.6	233	70.4	237	67.3	175	59.1
<Daily	677	32.2	133	35.7	156	43.1	57	28.9	27	13.9	96	29.0	107	30.4	101	34.1
Daily	94	4.5	25	6.7	31	8.6	7	3.6	1	0.5	2	0.6	8	2.3	20	6.8
Recreational drug use																
None	1970	93.6	370	99.2	309	85.4	191	97.0	194	100.0	326	98.5	350	99.4	230	77.7
Marijuana	65	3.1	1	0.3	24	6.6	3	1.5	0		2	0.6	2	0.6	33	11.2
Khat	97	4.6	0		45	12.4	5	2.5	0		0		0		47	15.9
Other*	6	0.3	2	0.5	0		0		0		3	0.9	0		1	0.3

° Range of values from the 2.5^th^ %ile to 97.5^th^ %ile

* Includes cocaine and off-label use of prescription medications.

### Hematology study consensus intervals

**Figure 1:**
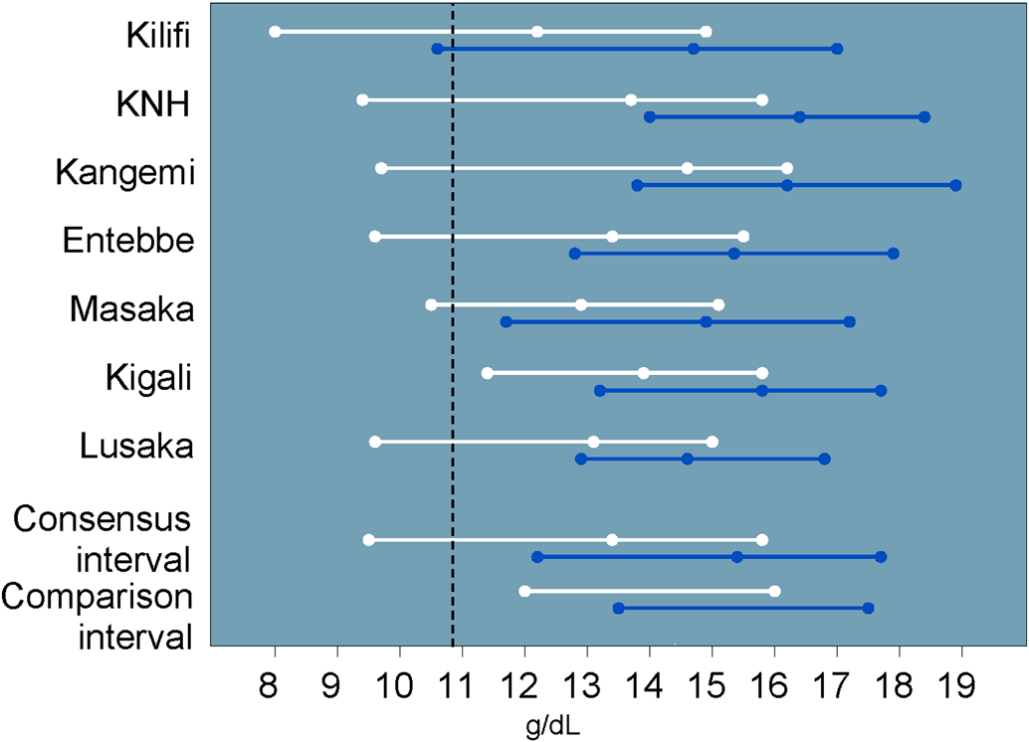
Hemoglobin intervals and medians by site and gender (men: blue, women: white) including U.S.-based comparison interval and cutoff for DAIDS grade one severity (vertical dashed line).

The hematology results are shown in Table 2. Due to significant gender and site variability, the complete data set could not be used to construct study consensus intervals for four of the 12 analytes (hematocrit, RBC, eosinophils and basophils). Women had lower hematocrit (median 39.7% vs. 45.1%, p<0.001) and lower hemoglobin values than men (median 13.4 vs. 15.4 g/dL, p<0.001) (Figure 1). Median hematocrit values for men and women at Kangemi and men at KNH were significantly higher than the study consensus interval and were therefore not included in the consensus interval (for details, see complete study report). In general, hematocrit values (and to a lesser degree, hemoglobin) tended to be lower than the U.S.-based comparison interval. Over 20% (187/846) of women had hematocrit values that were OOR (mostly lower) versus the U.S.-based comparison interval.

**Table 2:** Hematology results, U.S.-based comparison intervals and out of range (OOR) values

					OOR‡	
Analytes	N	Study Consensus interval	Units	U.S.-based Comparison interval*	N	%
Hemoglobin						
Male	1083	12.2–17.7	g/dL	13.5–17.5	140	12.9
Female	1022	9.5–15.8	g/dL	12.0–16.0	169	16.5
Hematocrit 1						
Male	799	35.0–50.8	%	41–53	151	18.9
Female	846	29.4–45.4	%	36–46	187	22.1
RBC^2^	1929	3.8–6.2	×10^6^ cells/µL	NA		
Male	1083	4.0–6.4	×10^6^ cells/µL	4.5–5.9	231	21.3
Female	846	3.8–5.6	×10^6^ cells/µL	4.0–5.2	141	16.7
MCV	2105	68–98	fl	80–100	403	19.1
Platelets	2105	126–438	×10^3^ cells/µL	150–350	360	17.1
Total WBC	2105	3.1–9.1	×10^3^ cells/µL	4.5–11.0	602	28.6
Neutrophil count	2103	1.0–5.3	×10^3^ cells/µL	1.8–7.7	604	28.7
Neutrophil (%)	2103	25–66	%	40–70	721	34.3
Lymphocyte count	2105	1.2–3.7	×10^3^ cells/µL	1.0–4.8	18	0.9
Lymphocyte (%)	2105	23–59	%	22–44	798	37.9
Monocytes count	2103	0.20–0.78	×10^3^ cells/µL	0–0.8	41	2.0
Monocytes (%)	2103	4.5–13.1	%	4–11	181	8.6
Eosinophils count	2104	0.04–1.53	×10^3^ cells/µL	0–0.45	437	20.8
Eosinophils (%) 3	1921	0.8–21.8	%	0–8	361	18.8
Basophils count 4	1750	0.01–0.15	×10^3^ cells/µL	0–0.2	22	1.3
Basophils (%) 5	1429	0.4–2.5	%	0–3	26	1.8
CD4 count	2100	457–1628	cells/µL	518–1981	109	5.2
CD8 count	2100	230–1178	cells/µL	270–1335	146	7.0

* [22], except differential counts [23] and CD4/CD8 counts [Beckton Dickson package insert]

‡ The number and percent of African values outside the U.S.-based comparison interval

1 Excludes men from Kangemi and KNH, and women from Kangemi

2 Excludes women from Kangemi

3 Excludes men from Masaka

4 Excludes all Lusaka volunteers

5 Excludes all Lusaka and Entebbe volunteers, and women from Kilifi

**Figure 2:**
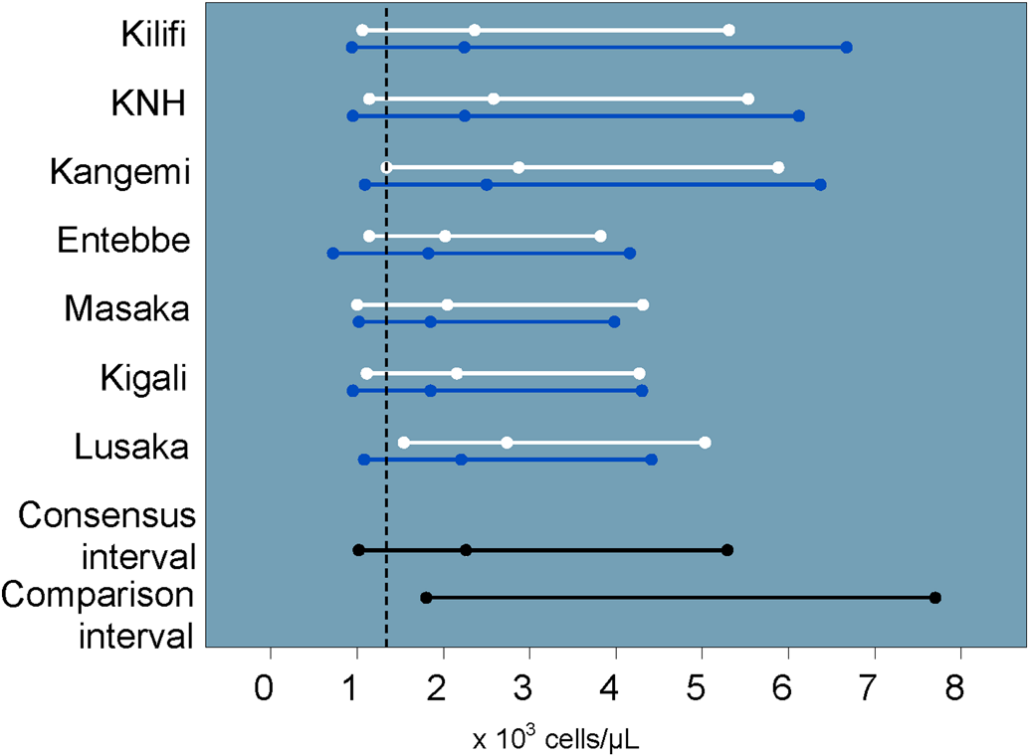
Neutrophil intervals and medians by site and gender (men: blue, women: white) including U.S.-based comparison interval and cutoff for DAIDS grade one severity (vertical dashed line).

**Figure 3:**
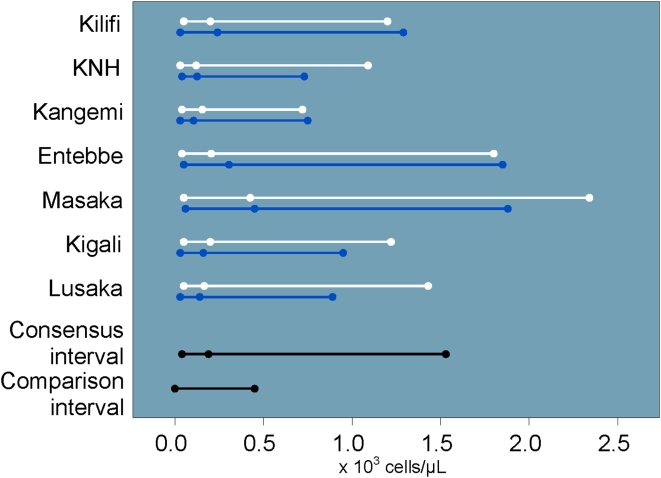
Eosinophil count intervals and medians by site and gender (men: blue, women: white) including U.S.-based comparison interval.

There was no significant gender or site variability for complete WBC counts across our study populations. The study consensus interval was lower than the U.S.-based comparison interval with nearly 29% of values OOR. Neutrophil counts also tended to be low (Figure 2), with a similar proportion of OOR values. Eosinophil values tended to be high (Figure 3). The eosinophil counts did not vary significantly by site or gender. Because eosinophil percent were significantly higher among men in Masaka, these values were not included in the study consensus interval. Basophil counts and percent varied by site, and the study consensus intervals do not include values from Lusaka. The study consensus interval for basophil percent also excludes values from Entebbe and women from Kilifi.

**Figure 4:**
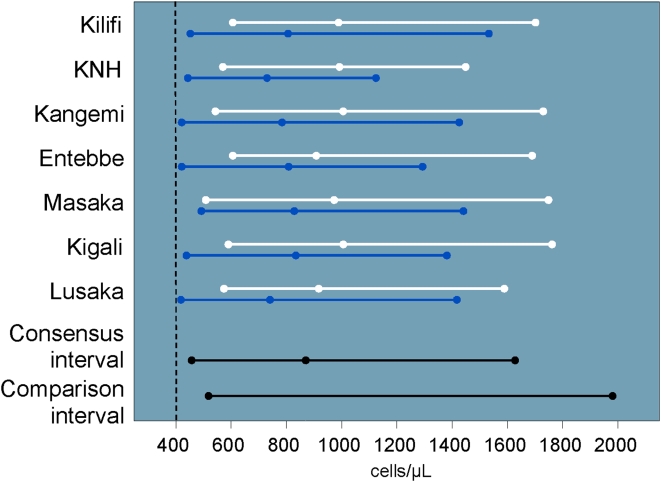
CD4 count intervals and medians by site and gender (men: blue, women: white) including U.S.-based comparison interval and cutoff for DAIDS grade one severity (vertical dashed line).

There were no significant site or gender differences in CD4 (Figure 4) or CD8 T cell intervals. Overall, the study consensus interval for CD4 T cell counts was similar to the U.S.-based comparison interval at 457 to 1640 cells/µL. Eight volunteers had CD4 T cell counts ≥2000 cells/µL and were coded as 2000 cells/µL. Nine volunteers had CD4 T cell counts <350 cells/µL, (range: 160–333 cells/µL) none of whom had antibody or PCR evidence of HIV infection. Few volunteers had values that were OOR for CD4 (5.2%) or CD8 (7.0%) T cell counts (Table 2).

### Chemistry study consensus intervals

**Figure 5:**
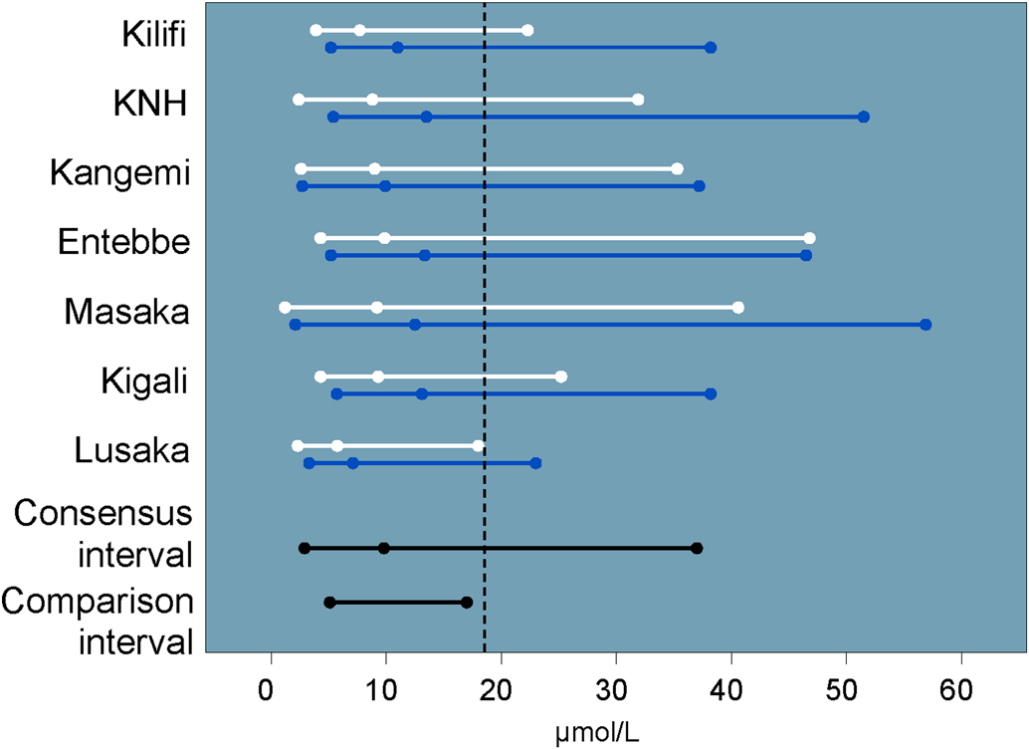
Total bilirubin intervals and medians by site and gender (men: blue, women: white) including U.S.-based comparison interval and cutoff for DAIDS grade one severity (vertical dashed line).

**Figure 6:**
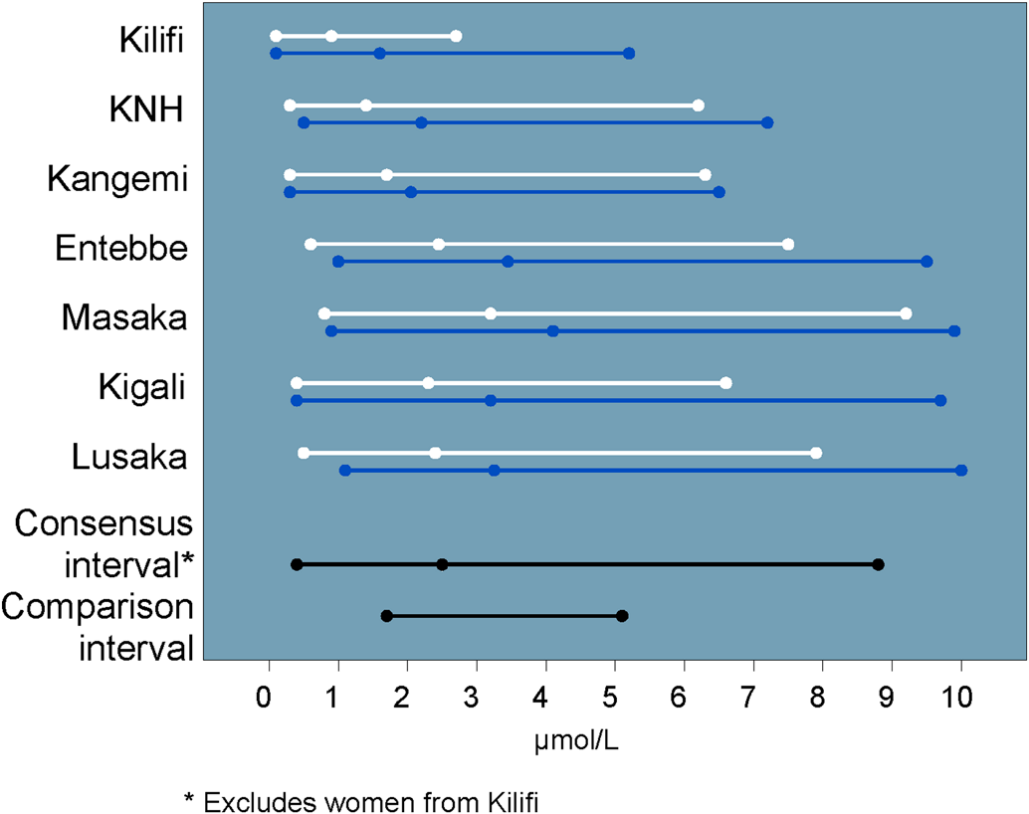
Direct bilirubin intervals and medians by site and gender (men: blue, women: white) including U.S.-based comparison interval.

The chemistry results are shown in Table 3. Due to significant gender and site variability, we were unable to create study consensus intervals using the complete data set for three of the 12 analytes (direct bilirubin, total immunoglobulin gamma [IgG], and lactate dehydrogenase [LDH]). Our total and direct bilirubin study consensus intervals were considerably wider than the U.S.-based comparison intervals; 31% and 42% of study values were OOR, respectively (Figures 5 and 6). The direct bilirubin interval from women in Kilifi was significantly lower than the other intervals from other sites, so values from women in Kilifi were excluded from the study consensus interval.

**Table 3:** Chemistry results, U.S.-based comparison intervals, and out of range (OOR) values

Analytes	N	Study Consensus interval	Units	U.S.-based Comparison interval*	OOR‡	
					N	%
Creatinine	2103	47–109	µmol/L	0–133	3	0.1
AST (SGOT)	2103	14–60	IU/L	0–35	244	11.6
ALT (SGPT)	2103	8–61	IU/L	0–35	248	11.8
Bilirubin direct 1	1906	0.4–8.8	µmol/L	1.7–5.1	792	41.6
Bilirubin total	2102	2.9–37.0	µmol/L	5.1–17	651	31.0
Total IgG 2	1919	759–2776	mg/dL	614–1295	1594	83.1
LDH 3	1674	214–528	IU/L	100–190	1663	99.3
Amylase	2103	35–159	IU/L	60–180	686	32.6
ALP 4	1021	48–164	IU/L	30–120	142	13.9
CPK	2101	53–552	IU/L	NA		
Male	1080	60–709	IU/L	60–400	119	11.0
Female	1021	49–354	IU/L	40–150	290	28.4
Albumin	2103	35–52	g/L	35–55	41	2.0
Total protein	1772	58–88	g/L	55–80	290	16.4

* [22]

‡ The number and percent of African values outside the U.S.-based comparison interval

1 Excludes women from Kilifi

2 Excludes men from Masaka

3 Excludes all Masaka volunteers and males from KNH

4 No data available from KNH, Kangemi, Entebbe or Masaka

**Figure 7:**
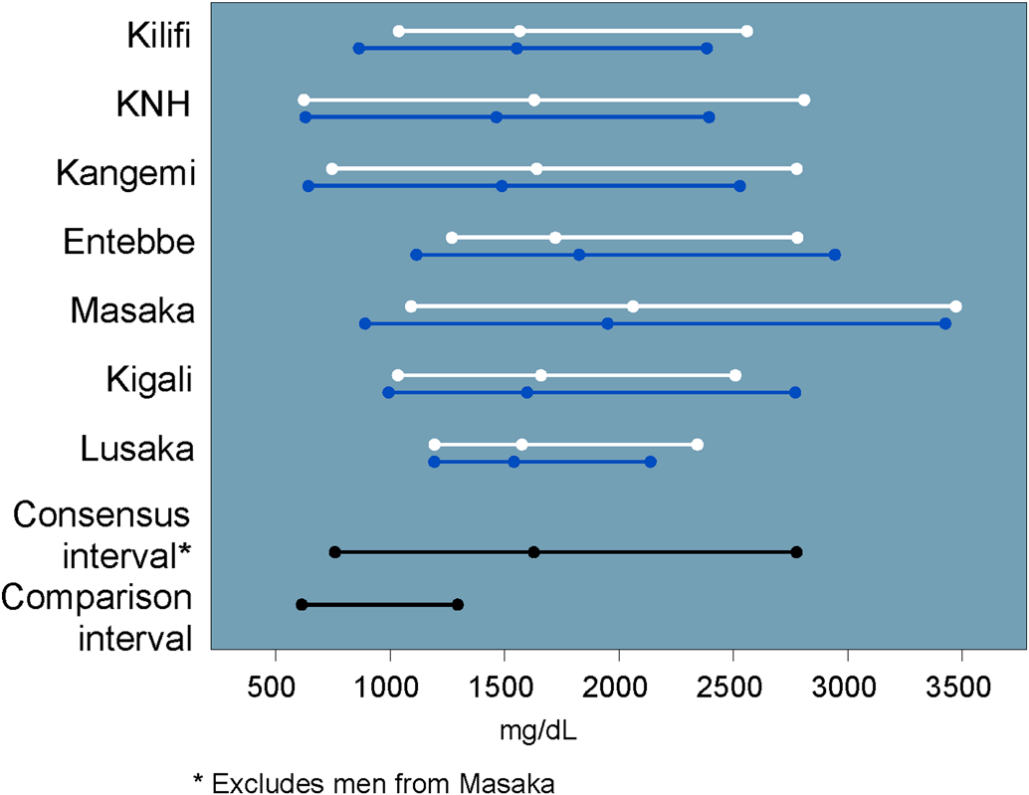
IgG intervals and medians by site and gender (men: blue, women: white) including U.S.-based comparison interval.

**Figure 8:**
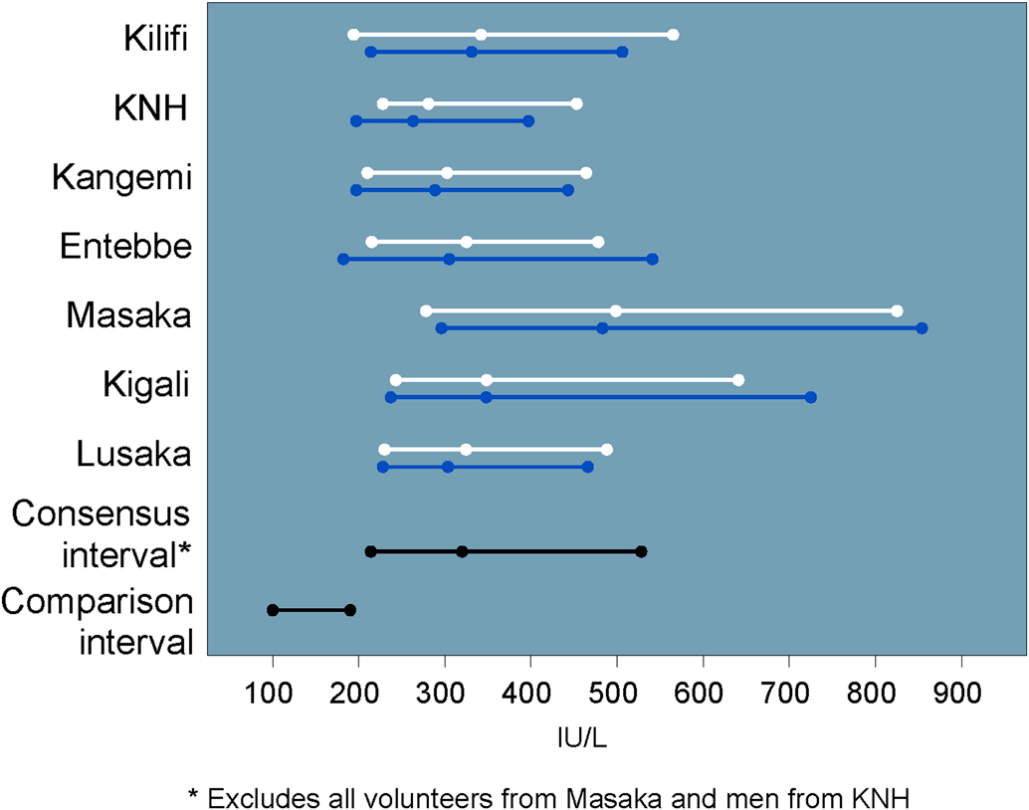
LDH intervals and medians by site and gender (men: blue, women: white) including U.S.-based comparison interval.

Values for IgG, creatinine phosphokinase (CPK) and LDH tended to be higher than their respective U.S.-based comparison intervals. In particular, 83% of IgG values and >99% of LDH values were OOR versus the U.S.-based comparison intervals. The study consensus interval for IgG excluded values from men in Masaka (Figure 7), and the study consensus interval for LDH excluded all Lusaka volunteers and men from KNH (Figure 8).

The study consensus interval for amylase was 35–159 IU/L, compared with 60–180 IU/L in the U.S., and there were no significant differences by site or gender. Both aspartate aminotransferase (AST, or SGOT) and alanine aminotransferase (ALT, or SGPT) intervals were slightly higher than their respective U.S.-based comparison intervals. Kangemi men had the highest levels of both AST and ALT, and the highest reported rates of alcohol intake were from this site (9% drink daily, 43% drink less than daily).

### “Adverse Events” by U.S.-Derived Grading Criteria

When the DAIDS AE criteria were applied to the 12 analytes evaluated in our study for which applicable values exist, a total of 744/2105 (35.3%) volunteers would have been considered to have had at least one laboratory-based AE: 319 (15.2%) with a hematology AE and 511 (24.3%) with a chemistry AE (Table 4). Had this clinically healthy study population been in an actual clinical trial, 3 volunteers would have been classified as having a grade 4 (“life threatening”) AE: one due to low hemoglobin and two due to elevated total bilirubin results. The prevalence of laboratory “AEs” varied by analyte (range: 0–15%). Among women, 67 (6.6%) hemoglobin values would have been classified as an AE: 33 (3.2%) grade 1, 17 (1.7%) grade 2, 16 (1.6%) grade 3, and 1 (0.1%) grade 4. Study consensus intervals for neutrophil results tended to be lower than the U.S.-based comparison interval, and 201 (9.6%) volunteers would have been classified as a grade 1 or higher AE. Of these, 38 (1.8%) would have been classified as grade 2 and 7 (0.3%) considered grade 3. Study AST values also tended to be higher than the U.S.-based comparison intervals with the following results: 103 (4.9%) grade 1, 20 (1.0%) grade 2, and three (0.1%) grade 3. Study ALT values also tended to be high classifying 132 AEs: 120 (5.7%) grade 1, 10 (0.5%) grade 2 and two (0.1%) grade 3. Total bilirubin was the analyte with the greatest difference from U.S.-based values, with nearly 15% of volunteers who would have been classified as having an AE: 191 (9.1%) grade 1, 93 (4.4%) grade 2, 28 (1.3%) grade 3, and 2 (0.1%) grade 4.

**Table 4:** Analyte results and frequency of “adverse events” graded against western-derived DAIDS AE cutoffs*

				Grade 1			Grade 2			Grade 3			Grade 4		
Analytes	N	Study Consensus interval	Units	Cutoff	N	%	Cutoff	N	%	Cutoff	N	%	Cutoff	N	%
Hemoglobin															
Male	1083	12.2–17.7	g/dL	≤10.9	2	0.2	≤9.9	1	0.1	≤8.9	3	0.3	≤7.0	0	0
Female	1022	9.5–15.8	g/dL	≤10.9	33	3.2	≤9.9	17	1.7	≤8.9	16	1.6	≤7.0	1	0.1
Platelets	2105	126–438	×10̅3 cells/µL	≤124.9	28	1.3	≤99.9	18	0.9	≤49.9	5	0.2	≤25	0	0
WBC	2105	3.1–9.1	×10̅3 cells/µL	≤2.5	6	0.3	≤1.9	0	0	≤1.49	0	0	≤1	0	0
Neutrophil count	2103	1.0–5.3	×10̅3 cells/µL	≤1.3	156	7.4	≤0.99	38	1.8	≤0.749	7	0.3	≤0.5	0	0
Lymphocyte count	2105	1.2–3.7	×10̅3 cells/µL	≤0.65	0	0	≤0.59	0	0	≤0.49	0	0	≤0.35	0	0
CD4	2100	457–1628	cells/µL	≤400	11	0.5	≤299	3	0.1	≤199	1	0.1	≤100	0	0
Creatinine	2103	47–109	µmol/L	≥146.3	0	0	≥186.2	0	0	≥252.7	0	0	≥465.5	0	0
AST (SGOT)	2103	14–60	IU/L	≥43.8	103	4.9	≥91.0	20	1.0	≥178.5	3	0.1	≥350.0	0	0
ALT (SGPT)	2103	8–61	IU/L	≥43.8	120	5.7	≥91.0	10	0.5	≥178.5	2	0.1	≥350.0	0	0
Bilirubin total	2102	3.9–37.0	µmol/L	≥18.7	191	9.1	≥27.2	93	4.4	≥44.2	28	1.3	≥85.0	2	0.1
Albumin	2103	35–52	g/L	≤35.0	52	2.5	≤29.0	1	0.1	≤20	0	0	NA		
CPK															
Male	1080	60–709	IU/L	≥1200	7	0.7	≥2400	1	0.1	≥4000	2	0.2	≥8000	0	0
Female	1021	49–354	IU/L	≥450	9	0.9	≥900	2	0.2	≥1500	0	0	≥3000	0	0

* Chemistry cutoffs [16] derived from [22]. Hemoglobin, platelets, WBC, neutrophil, lymphocyte and CD4 counts provided in [16]

## Discussion

As the attention of the global health community increasingly turns to the development of preventive and therapeutic interventions for HIV, tuberculosis and malaria, among others, Africa has become an important venue for clinical trials [1], [24]. A thorough understanding of the health status of potential clinical trial volunteers in Africa is essential for planning and executing such trials in a safe, efficient and ethical manner. In this regard, it is essential to use locally appropriate laboratory reference intervals to assess volunteer health, monitor laboratory-based AEs, and assure that healthy individuals who want to volunteer to participate in clinical trials are not unnecessarily prevented from participating if it is safe for them to do so.

Our aim was to assess “normal” reference intervals in healthy, HIV-uninfected adults most representative of the population likely to enroll in future HIV prevention clinical trials and to compare them to U.S.-derived reference intervals. Discussion about how to define “normal” in the context of physiologic variation is far from new: it has been debated since the seminal studies of Claude Bernard in the 19^th^ century [25]. In defining “normal” for clinical trial participation, it seems desirable to study persons whose robust health minimizes any concerns that an investigational product or clinical procedure will cause harm, or that disease will distort the results of the trial. Hence, this study excluded participants whose medical history or physical examination indicated they were unwell. By both their own judgment and the clinical trial physicians' evaluation, the participants in this study were healthy. Application of reference intervals derived from populations of different ethnicity and environment might appear to be more conservative, but the primary question of trials in Africa is, and should be, *will this investigational product be safe and efficacious in this population?* To most efficiently answer that question, we believe that reference intervals derived from African populations should be used in selecting participants and evaluating adverse events in future African research.

We found that several analytes that are used as inclusion criteria for clinical trials differed significantly from the U.S.-based comparison intervals. Study volunteers whose values for certain analytes, particularly neutrophil counts and total bilirubin, fall on the outer edge of the study consensus interval would be classified as having a DAIDS Grade 1 or 2 adverse event despite being clinically healthy adults. If such volunteers do not participate in future preventive clinical trials, the trial results would be difficult to generalize to the healthy population as a whole. The U.S. Food and Drug Administration (FDA) recently created a toxicity assessment scale [26] similar to that provided by DAIDS [16] but specifically for use with preventive vaccine clinical trials. While the grading criteria are very similar, the FDA grades analytes that DAIDS does not (e.g. WBC and eosinophil counts), and it grades some analytes more conservatively than DAIDS (e.g. CPK, lymphocyte counts, neutrophil counts). Evaluating our data against the newer FDA guidelines would classify an even greater proportion of our healthy volunteers than we report here as being ineligible for studies due to having an “adverse event”. Of note, the FDA document does highlight the importance of considering locally appropriate clinical reference values when grading AEs [26].

Hemoglobin, hematocrit and RBC values tended to be lower in our study than in the U.S.-based comparison intervals. Similar findings have previously been observed in healthy, HIV-uninfected populations in Uganda [11]. Lower hemoglobin levels have also been reported in Ethiopia [9]. Possible explanations include poor nutritional status resulting in iron deficiency, genetic disorders (e.g. thalassemia, sickle cell trait), or infection with helminths or other parasites (e.g. malaria or schistosomiasis) for which we did not test. Pregnancy and childbirth may affect a woman's hematologic profile. These effects are largely transient, and we excluded from analysis the data from pregnant women. It is important to include healthy women in trials, given difficulties in recruiting and retaining women in clinical trials [27]. When screening volunteers for clinical trial participation, investigators must be aware that repeated phlebotomy from clinical trial participation may transiently decrease hemoglobin by as much as 1.0gm/dL [28]. Conversely, altitude can increase these hematologic parameters. Participants at the two study centers in Nairobi had higher hematocrit and RBC; these centers are at an elevation of 1680 meters and are the only centers that are not in a malaria-endemic area. The KNH study population was primarily drawn from medical students and clinicians, whose higher level of education and socioeconomic status, and presumed better health status, could also contribute to the hematological indices that differed significantly from other African research centers warranting exclusion from the consensus intervals per CLSI guidelines, Depending on the location of the clinical research center, there may also be seasonal variation in hematologic values due to malaria. However a preliminary report on data collected from a substudy across rainy and dry seasons in Kigali suggests that any seasonal changes in analyte values are modest and of limited clinical significance [29].

Lower neutrophil counts compared to western reference intervals have been reported in African populations [5], [10], [11], [30] and among blacks in the U.S. [15]. Our study consensus interval for neutrophil counts was lower than the U.S.-based comparison interval, and nearly 10% of our study population would have been classified as having a neutrophil count related AE. The lower neutrophil count may reflect genetic and/or environmental differences. We also found that basophils and eosinophils in both genders were elevated relative to the U.S.-based comparison intervals, likely due to a high prevalence of parasitic infections in the study population and exposure to a broader range of environmental antigens [31], [32]. We did not perform malaria blood smears on asymptomatic study volunteers. Malaria and other parasitic infestations can induce eosinophilia. Malaria is endemic at all participating study sites except Nairobi. Kangemi and KNH volunteers had among the lowest eosinophil counts at all the sites (Figure 3), although the difference was not sufficient to exclude them from the consensus intervals. Preliminary results from a sub-study examining stool for ova and parasites in Lusaka, Entebbe and Kigali demonstrate the presence of ova or parasites in as many as one third of stool samples (data not shown).

Studies in Uganda [13] and Tanzania [14] suggest that modest differences across genders and between Western and African values may exist for CD4 counts. One well-controlled study found lower CD4 counts among 142 healthy HIV-uninfected Ethiopians compared to 1,356 Dutch control volunteers [9]. Our study did not demonstrate significant site or gender differences in CD4 T cell counts, and our study consensus interval was similar to the U.S.-based comparison interval. The different conclusions may be due to the fact that the Ethiopian-Dutch study did not employ CLSI guidelines to compare CD4 counts across study populations. With sufficient sample sizes, standard statistical comparisons (e.g., student's t test, Wilcoxon rank sum) may detect a statistically significant difference where the clinical significance is questionable [21].

We found a greater proportion of OOR values in clinical chemistry results than in hematology results. The highest prevalence of OOR values was found in LDH, IgG, and direct bilirubin. Neither LDH nor IgG are included in the DAIDS AE tables or the FDA guidelines for toxicity grading [16], [26]. LDH is a non-specific laboratory marker that can be elevated in several common disorders or due to hemolysis during specimen collection and processing. Although we did not collect data on specimen quality for analysis, the study laboratories operated under quality assurance programs to minimize issues with sample collection. The most common possible causes of asymptomatic LDH elevation relevant to our study population include anemia, skeletal muscle trauma due to physical exertion, and asymptomatic liver disease. Although we did not fractionate the LDH, we did not see clinically significant correlations between elevated LDH and elevated hepatic transaminases or indicators of anemia such as lower hemoglobin and hematocrit (data not shown). Although 99% of the LDH values observed in this study were outside the U.S.-based comparison interval, the lower limits of our study consensus intervals did overlap with the reference intervals from the Washington Manual (100–250 IU/L) [33] and Bakerman's ABCs (118–273 IU/L for men and 122–220 IU/L for women) [23]. Such a systematic difference in values for LDH may suggest a difference in laboratory methods. However this cannot be confirmed because the reference texts for the U.S.-based comparison intervals do not provide laboratory methodologies. The elevated IgG may be due to the larger burden of immunological challenges associated with infectious agents prevalent in a tropical environment and/or a developing country [31], [32].

The highest proportion of events that would be classified as AEs in a clinical trial was observed in total bilirubin. Possible explanations for an elevated total bilirubin in our study population include hemolysis secondary to malaria or sickle cell trait, malnutrition, physical exertion, and cirrhosis, although conditions such as Gilbert's syndrome, which has been reported in South Africa [34], cannot be ruled out. High levels of reported alcohol intake were not common in this population, and volunteers with jaundice or laboratory evidence of chronic hepatitis B or C were excluded from enrollment and analysis. We also observed a high frequency of elevated direct bilirubin that varied by location and gender. The causes of elevated direct bilirubin are frequently related to hepatocellular disease, biliary tract obstruction, and inherited syndromes which usually have clinical manifestations. However, the population was carefully screened by history and physical examination, and we did not see a correlation between either AST or ALT and total or direct bilirubin in asymptomatic volunteers (data not shown). Previous IAVI-sponsored Phase I HIV vaccine trials in Kenya and Uganda found high bilirubin levels in several healthy volunteers with no concurrent elevation in transaminases [35] Therefore, we feel hepatic disease is an unlikely explanation for the many OOR chemistry liver function tests in our study populations. The role of environmental factors remains unclear.

The approach to defining consensus reference intervals is complex. We have chosen to apply the CSLI method for combining values from different groups. As recommended by the CLSI guidelines, we did not censor outliers from our data set from volunteers who were otherwise eligible for inclusion. The results presented include three clinically healthy individuals whose laboratory values for hemoglobin and total bilirubin would have been considered as Grade 4 AE (“potentially life threatening”) according to DAIDS grading criteria. Excluding outliers is discussed by Horn *et al.* with regards to hospital-generated ranges [36], [37], where the likelihood of including individuals with disease is high. Our study populations were carefully screened to exclude unwell persons, and clinical reference intervals based on ranked observations and with our sample size are not significantly affected by the inclusion of potential outliers [21]. Additionally, methods to identify and censor outliers remain unsatisfactory [38].

Given the large sample size at each research center, we found numerous statistically significant (p<0.05) differences across sites and gender that did not merit excluding those populations from the consensus intervals. Sub-group comparisons based on the differences in means rather than the interval endpoints themselves may affect the final consensus intervals. We found, for example, that while we were able to create a consensus interval for neutrophils from all sites, the upper limits of the Kenyan intervals were consistently higher than what we observed at other sites (Figure 2). While the CLSI guidelines do consider interval variability to some degree, the significance of creating a consensus reference interval without a direct comparison of the upper and lower limits is unclear. A possible remedy might include regression analysis performed on percentiles, rather than only on the mean value (e.g., the SAS QUANTREG procedure).

Furthermore, the CLSI guidelines do not provide clear guidelines on how to proceed when comparing multiple sub-groups. We chose to do a stepwise analysis as described in the methods. This was first performed separately by site, then for males and females. Another approach might be to combine the data from sites in no particular order; or to first combine data from all sites, then to remove one site at a time (i.e., backwards elimination). With multiple sites under study, these approaches might result in different or multiple distinct groups. These observations will be the basis of future work and suggest that the CLSI guidelines may need to be revisited if it is desired to establish region-wide ranges.

Our study populations were selected on the basis of their willingness to receive VCT and participate in HIV-related research. This potential selection bias limits the ability to generalize these results to the entire adult population of the region. Although we were able to create study consensus intervals for the majority of the analytes measured, some analyte intervals were not compatible across study sites or populations. This further underscores the importance of using locally appropriate laboratory reference intervals for public health and research studies. For those research centers without locally-derived reference intervals, a regionally-derived consensus interval and sound clinical judgment may be the next best alternative until more data become available. In all situations, the volunteer participant's health and safety is of utmost priority.

### Conclusions

Clinical trials of interventions intended for use in Africa should enroll healthy persons who are representative of the population that will receive the intervention. Exclusion of eligible volunteers based on reference ranges derived from a different population creates unnecessary delays in enrolment. Applying non-local reference intervals for hematology and biochemistry screening and grading of AEs would have excluded over one-third of these healthy Africans from trial participation. Furthermore, the use of non-local laboratory reference intervals in clinical research may ultimately compromise the scientific validity of clinical trial conclusions by selecting participants that are not representative of the source population. This work successfully created a set of consensus intervals for the majority of analytes studied, however local conditions such as elevation, endemic diseases, and nutritional factors must be taken into account. These study findings on laboratory intervals from healthy African volunteers will be useful in the design, conduct, and evaluation of future clinical trials and highlight the need for local data.
